# A systematic review and meta-analysis on effects of aerobic exercise in people with Parkinson’s disease

**DOI:** 10.1038/s41531-022-00418-4

**Published:** 2022-10-31

**Authors:** Kai Zhen, Shiyan Zhang, Xifeng Tao, Gen Li, Yuanyuan Lv, Laikang Yu

**Affiliations:** 1 Key Laboratory of Physical Fitness and Exercise, Ministry of Education, Beijing Sport University, Beijing, China; 2 Department of Sports Performance, Beijing Sport University, Beijing, China; 3 China Institute of Sport and Health Science, Beijing Sport University, Beijing, China

**Keywords:** Parkinson's disease, Quality of life

## Abstract

Previous studies have shown that aerobic exercise is an effective way to improve symptoms of Parkinson’s disease (PD). The aim of this study [PROSPERO CRD42022340730] was to explore the effects of aerobic exercises on balance, gait, motor function, and quality of life in PD patients. Searches were performed in PubMed, Web of Science, and EBSCO electronic databases. The Cochrane risk assessment tool was used to evaluate the methodological quality of the included literature. From 1287 search records initially identified, 20 studies were considered eligible for systematic review and meta-analysis. There was a significant effect of aerobic exercise on improving timed up and go test [standardized mean difference (SMD), −0.41 (95% CI, −0.61 to −0.22), *p* < 0.00001], Berg Balance Scale [0.99 (95% CI, 0.76 to 1.23), *p* < 0.00001], stride/step length [0.32 (95% CI, 0.03 to 0.61), *p* = 0.03], gait velocity [0.49 (95% CI, 0.20 to 0.78), *p* = 0.0009], Unified Parkinson’s Disease Rating Scale Part-III [-0.40 (95% CI, −0.55 to −0.24), *p* < 0.00001], and 6-minute walking test [0.35 (95% CI, 0.13 to 0.56), *p* = 0.002] in people with PD, but not in step cadence [−0.08 (95% CI, −0.43 to 0.27), *p* = 0.65] and Parkinson’s Disease Questionnaire-39 [−0.113 (95% CI, −0.39 to 0.13), *p* = 0.32]. Aerobic exercise had beneficial effects in improving balance, gait (velocity and stride/step length), and motor function in PD patients. However, aerobic exercise had no significant associations with the step cadence and quality of life in PD patients.

## Introduction

Parkinson’s disease (PD) is a progressive neurodegenerative disorder commonly affecting older adults worldwide^1^. The manifestations of PD primarily include dysfunction of the somatomotor system (such as rigidity, bradykinesia, postural instability, gait dysfunction, and tremor)^2,3^. The disease course is usually accompanied by impairment of non-motor functions (such as dementia, hyposmia, and gastrointestinal alterations)^4–6^. The dysfunction caused by PD deprives patients of their ability to perform daily life activities and thus their independence^7^. Currently, PD affects about 0.3% of the general population and 1–3% of the over-65 population, whose numbers will increase from 8.7 million to 9.3 million by 2030^8,9^.

PD is usually treated with medication (levodopa, dopamine agonizts) and surgery (deep brain stimulation)^10^. Pharmacotherapy is a common treatment approach that includes the optimization of levodopa-containing formulations and multiple drug dosing. However, as motor symptoms (dyskinesia, freezing) worsen, patients have a shorter and more limited response to treatment (progressively weaker), requiring frequent doses of levodopa and its agonizts in advanced stages, and surgical intervention is also accompanied by safety concerns and side effect. Significant improvements in PD symptoms have been inconsistent after taking levodopa and related medications, and patients must face severe motor and cognitive impairments^11^. Conventional pharmacotherapy can also reduce an individual’s balance and walking ability and increase the risk of falls^12^. Additionally, two novel therapeutic approaches, neurotrophic factor therapy and cell transplantation, rely heavily on highly invasive stereotaxic surgery, so these strategies do not provide a perfect treatment for PD patients^13^.

There is an increasing emphasis on complementary and alternative treatments without medication, such as exercise. There is growing evidence that exercise has benefits on neuroplasticity and the ability of the brain to self-repair^14^. Animal studies have also found that exercise has a protective effect on the onset of PD symptoms^15^. Among the different types of exercise programmers, aerobic exercise is the most studied and is considered the best option for improving people’s health throughout the lifespan^16^. Studies have shown that aerobic exercise has neurorestorative and neuroprotective effects, possibly by regulating neurotrophic factors that support synaptogenesis and angiogenesis, inhibiting oxidative stress, and improving mitochondrial function^17^. Aerobic exercise, such as treadmill training, walking, and dance, can improve not only motor function but also gaits, balance, and quality of life in patients with moderate to severe PD^18–20^. Mehmet et al.^21^ reported that 14 patients with mild to moderate PD showed improvement in disease severity, balance, functional mobility, and upper extremity motor function after receiving stationary recumbent bicycle training. To our knowledge, Mak et al.^22^ investigated the effects of long-term (≥12 weeks) aerobic exercise on patients with PD, which included only eight randomized controlled trials (RCTs) and the number of included studies was quite small. Furthermore, due to the lack of short-term aerobic exercise, the effect of aerobic exercise on PD patients cannot be fully reflected. Therefore, further studies with more included studies are needed. Another study evaluated the effects of resistance training (RT), endurance training (ET), and other intensive exercise modalities (OITM) on patients with PD^23^. However, the authors included studies in which control group participants also received exercise interventions such as 60–80% heart rate reserve (HRR) voluntary exercise (two studies), and upper and lower limbs, stretching, and gait training (one study). In addition, one of the included studies used healthy adults as a control group, which may have had some impact on their findings. Therefore, we conducted a comprehensive systematic review and meta-analysis of RCTs to explore the effects of aerobic exercises on balance, gait, motor function, and quality of life in PD patients.

## Results

### Study selection

**Fig. 1:**
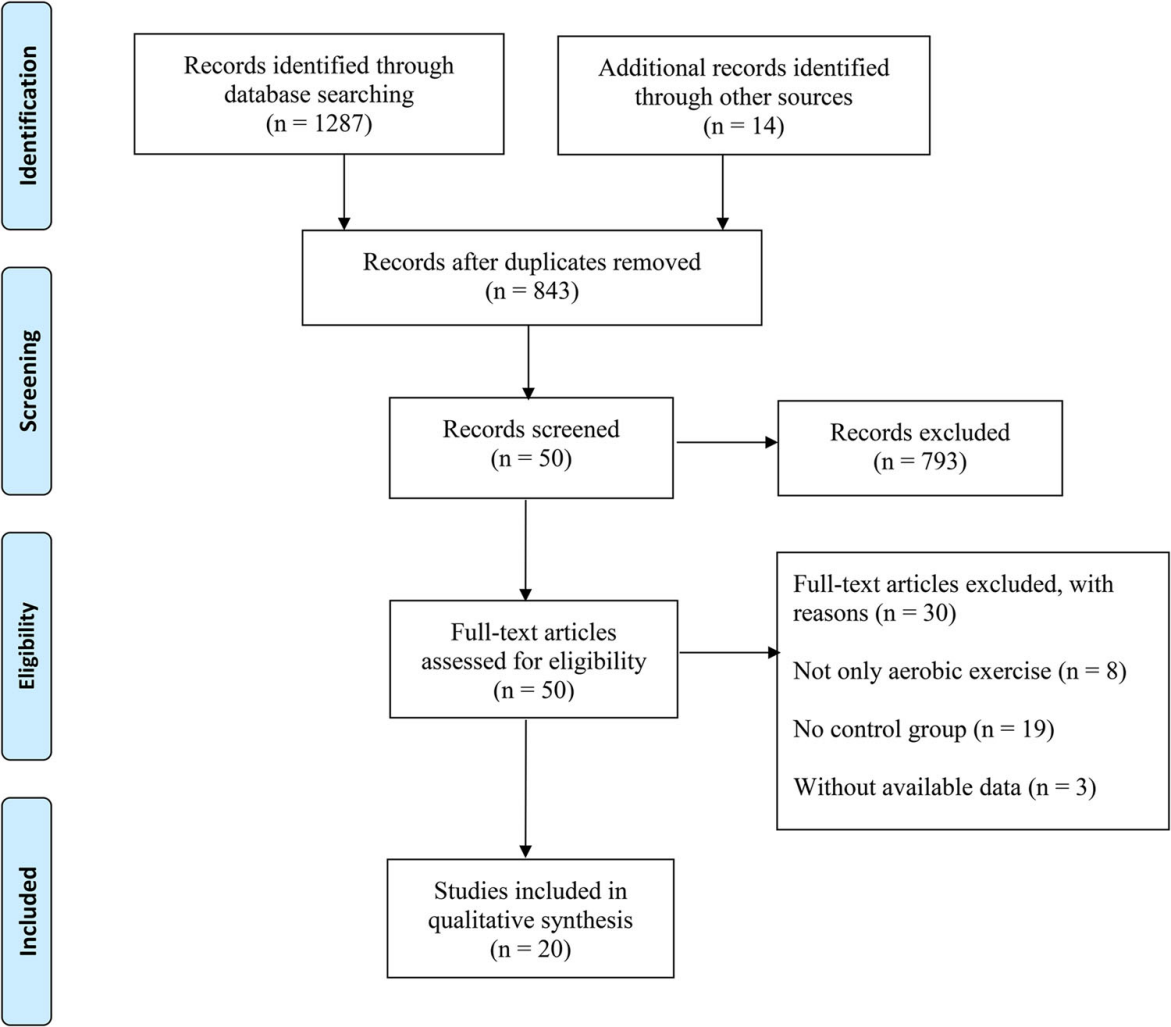
PRISMA flowchart of study selection.

As shown in Fig. 1, a total of 1287 search records were preliminarily retrieved, and 14 records were identified through other sources. After excluding the duplicates, 843 studies remained, and 793 studies were not eligible for inclusion through the title and abstract screening. Thirty studies were excluded by reading the full text of 50 studies: (1) the experimental group combined with other treatments (*n* = 8); (2) no control group (*n* = 19); and (3) the data could not be extracted (*n* = 3). Finally, 20 studies examining the effect of aerobic exercise on balance, gait, motor function, and quality of life in PD patients were considered eligible for systematic review and meta-analysis.

### Study characteristic

The main characteristics of the participants and interventions were shown in Table 1. The included studies involved 450 participants in the 23 exercise groups and 352 participants in the 20 control groups. Most participants with mild to moderate PD included 18 trials with 744 patients (Hoehn and Yahr stage I to III) and 2 trials with 58 patients (Hoehn and Yahr stage I to IV). All included studies reported aerobic interventions, such as treadmill training, walking, cycling, dancing, and other types of aerobic training. The control group had no other interventions, including usual care, normal daily activities, and conventional medication. Intervention duration ranged from 3 weeks to 6 months. Nine included studies used the BBS as the outcome^24–32^, and ten studies reported TUG data to assess the balance^24,28,30,31,33–38^. Thirteen studies reported the data from UPDRS-III, including 16 outcomes^24,27–29,31,33,36–42^. We extracted nine data for eight studies on 6MWT^24,28,31–33,35,39,42^, but only six and four trials reported data on gait^24,25,31,34,36,43^ and PDQ-39^33,37,39,42^, respectively.

**Table 1:** Characteristics of studies included in this meta-analysis.

Study	Country	Sample size	Age(y)	Stage of disease	Disease duration (y)	Intervention	Characteristics of Interventions	Outcome measures
Sage (2009)	NR	IG = 13 CG = 15	IG: 65.1 (9.3) CG: 68.6 (8.7)	UPDRS, IG: 22.2 (8.1) CG: 21.8 (7.2)	IG: 3.2 (2.9) CG: 2.5 (2.2)	IG: aerobic intervention CG: regular activity	30 min/session, 60–75% HRmax, two sessions per week for 10–12 weeks	UPDRS-III, TUG, Gait
Cannning (2012)	Australia	IG = 10 CG = 10	IG: 60.7 (5.9) CG: 62.9 (9.9)	Total: H&Y stage 1–3	IG: 6.1 (4.0) CG: 5.2 (4.1)	IG: treadmill walking CG: usual care	20-40 min/session, 60–80% of the average 6-min walk test speed, four sessions per week for 6 weeks	UPDRS-III, 6MWT, PDQ-39
Arfa-Fatollahkhani (2019)	Iran	IG = 11 CG = 9	IG: 60.63 (9.36) CG: 61.55 (8.57)	Total: H&Y stage 1.5–2.5	IG: 8.89 (5.14) CG: 8.50 (6.34)	IG: treadmill walking CG: usual care	30 min/session, 60%HRmax, two sessions per week for 10 weeks	TUG, 6MWT
Solla (2019)	Italy	IG = 10 CG = 10	IG: 67.8 (5.9) CG: 67.1 (6.3)	H&Y, IG: 2.1 (0.6) CG: 2.3 (0.4)	IG: 4.4 (4.5) CG: 5.0 (2.9)	IG: dance CG: usual care	90 min/session, two sessions per week for 12 weeks	UPDRS-III, 6MWT, BBS, TUG, Gait
Wan (2021)	China	IG = 20 CG = 20	IG: 64.95 (7.83) CG: 67.03 (7.47)	Total: H&Y stage 1–4	IG: 3.63 (1.52) CG: 3.25 (1.7)	IG: Qigong CG: regular medication	60 min/session, 70–80% HRmax, four sessions per week for 12 weeks	TUG, Gait
Hashimoto (2015)	Japan	IG = 15 CG = 14	IG: 67.9 (7.0) CG: 69.7 (4.0)	UPDRS, IG: 42.7 (13.9) CG: 33.4 (14.2)	IG: 6.3 (4.6) CG: 7.8 (6.2)	IG: dance CG: normal lives	60 min/session, one session per week for 12 weeks	BBS, TUG
Lee (2018)	Korea	IG = 25 CG = 16	IG: 65.8 (7.2) CG: 65.7 (6.4)	Total: H&Y stage 1–3	IG: 4.5 (3.3) CG: 4.4 (3.0)	IG: dance CG: normal lives	60 min/session, 60–75% HRmax, two sessions per week for 8 weeks	UPDRS-III, BBS
Hackney (2009)	US	IG-1 = 14 IG-2 = 17 CG = 17	IG-1: 68.2 (1.4) IG-2: 66.8 (2.4) CG: 66.5 (2.8)	Total: H&Y stage 1–4	IG-1: 6.9 (1.3) IG-2: 9.2 (1.5) CG: 5.9 (1.0)	IG-1: tango IG-2: waltz/foxtrot CG: no intervention	60 min/session, 60–75% HRR, two sessions per week, completing 20 sessions within 13 weeks	UPDRS-III, BBS TUG, 6MWT, Gait
Altmann (2016)	US	IG = 11 CG = 10	IG: 62.8 (8.6) CG: 67.8 (9.8)	Total: H&Y stage 1–3	NR	IG: aerobic exercise CG: normal activities	20–45 min/session, 50–75% HRR, three sessions per week for 16 weeks	UPDRS-III
Cugusi (2015)	Italy	IG = 10 CG = 10	IG: 68.1 (8.7) CG: 66.6 (7.3)	Total: H&Y stage 1–3	IG: 7.0 (2.0) CG: 7.0 (4.0)	IG: walking CG: conventional care	60 min/session, 60–80% HRR, two sessions per week for 12 weeks	UPDRS-III, BBS, TUG, 6MWT
Ganesan (2014)	US	IG = 20 CG = 20	IG: 57.6(9.1) CG: 59.1 (6.8)	Total: H&Y stage 1–3	IG: 5.7 (3.9) Cg: 5.5 (3.4)	IG: treadmill training CG: only stable dosage	30 min/session, four sessions per week for 4 weeks	UPDRS-III, BBS
Schenkman (2017)	US	IG-1 = 39 IG-2 = 42 CG: N = 38	IG-1: 64 (9) IG-2: 63 (10) CG: 64 (10)	Total: H&Y stage 1–2	Total: <5	IG-1: treadmill exercise IG-2: treadmill exercise CG: wait-list control	IG-1: 40–50 min/session, 80–85% HRmax, four sessions per week for 24 weeks IG-2: 40–50 min/session, 60–65% HRmax, four sessions per week for 24 weeks	UPDRS-III
Cakit (2007)	Turkey	IG = 21 CG = 10	Total: 71.8 (6.4)	Total: H&Y stage 2–3	Total: 5.58 (2.9)	IG: treadmill training CG: no intervention	30 ± 5 min/session, walking at the maximum speed, for 8 weeks	BBS
Protas (2005)	US	IG = 9 CG = 9	IG: 71.3(7.4) CG: 73.7 (8.5)	Total: H&Y stage 2–3	IG: 7.1 (5.1) CG: 8.1 (4.4)	IG: walking CG: no intervention	60 min/session, walking at the fastest, self-selected speed, three sessions per week for 8 weeks	Gait
Thaut (1996)	US	IG = 11 CG = 11	IG: 74.0 (3.0) CG: 71.0 (8.0)	IG: 2.6 (NR) CG: 2.5 (NR)	IG: 5.4 (3.0) CG: 8.5 (4.0)	IG: walking CG: normal daily activities	30 min/session, 60–120 steps/min, one session per week for 3 weeks	BBS, Gait
van der Kolk (2019)	Netherlands	IG = 65 CG = 11	IG: 59.3 (8.3) CG: 59.4 (8.3)	Mild stage Total: H&Y stage <2	IG: 15.1 (4.0) CG: 16.1 (4.5)	IG: aerobic exercise CG: active control group	30–45 min/session, 50–80% HRmax, three sessions per week for 24 weeks	UPDRS-III, TUG, 6MWT, PDQ-39
Nadeau (2014)	Canada	IG-1 = 11 IG-2 = 12 CG = 11	IG-1: 60.1 (6.8) IG-2: 64.0 (6.6) CG: 64.3 (5.6)	IG-1: 1.95 (0.15) IG-2: 1.92 (0.20) CG: 1.86 (0.23)	NR	IG-1: treadmill walking IG-2: treadmill walking CG: normal physical activity	60 min/session, 60–80% VO_2_peak, three sessions per week for 24 weeks	UPDRS-III, 6MWT, PDQ-39
Sacheli (2019)	Canada	IG = 20 CG = 15	IG: 66.76 (5.98) CG: 67.85 (8.50)	UPDRS, IG: 23.00 (10.42) CG: 26.77 (13.14)	IG: 3.91 (2.85) CG: 5.17 (4.26)	IG: aerobic group CG: control group	40–60 min/session, 60–80% VO_2_max, three sessions per week for 12 weeks	UPDRS-III, TUG
Li (2022)	China	IG = 20 CG = 20	IG: 67.57 (3.95) CG: 70.0 (5.59)	UPDRS, IG: 25.05 (17.45) CG: 26.79 (20.79)	IG: 6.93 (4.09) CG: 7.76 (4.55)	IG: Wuqinxi Qigong exercise CG: active control group	90 min/session, 60–70% HRmax or 6–12 on Brog scale, two sessions per week for 24 weeks	UPDRS-III, TUG, PDQ-39
Tollár (2019)	Netherlands	IG = 25 CG = 24	IG: 70.6 (4.10) CG: 67.5 (4.28)	H&Y, IG: 2.4 (0.51) CG: 2.4 (0.51)	IG: 7.5 (2.16) CG: 7.3 (2.21)	IG: cycling CG: a wait-listed control	60 min/session, 80% HRmax, five sessions per week for 5 weeks	BBS, 6MWT

*PD* Parkinson’s disease, *EG* experimental group, *IG* control group, *M* male, *NR* no report, *N* number, *H&Y* Hoehn and Yahr, *UPDRS* unified Parkinson’s disease rating, *UPDRS-III* unified Parkinson’s disease rating scale part-III, *TUG* timed up and go test, *BBS* Berg balance scale, *6MWT* 6-minute walking test; Data were presented as mean (standard deviation), *HRmax* maximum heart rate, *HRR* heart rate reserve, *VO*_*2*_*peak* peak oxygen uptake, *VO*_*2*_*max* maximal oxygen consumption.

### Risk of bias

**Fig. 2:**
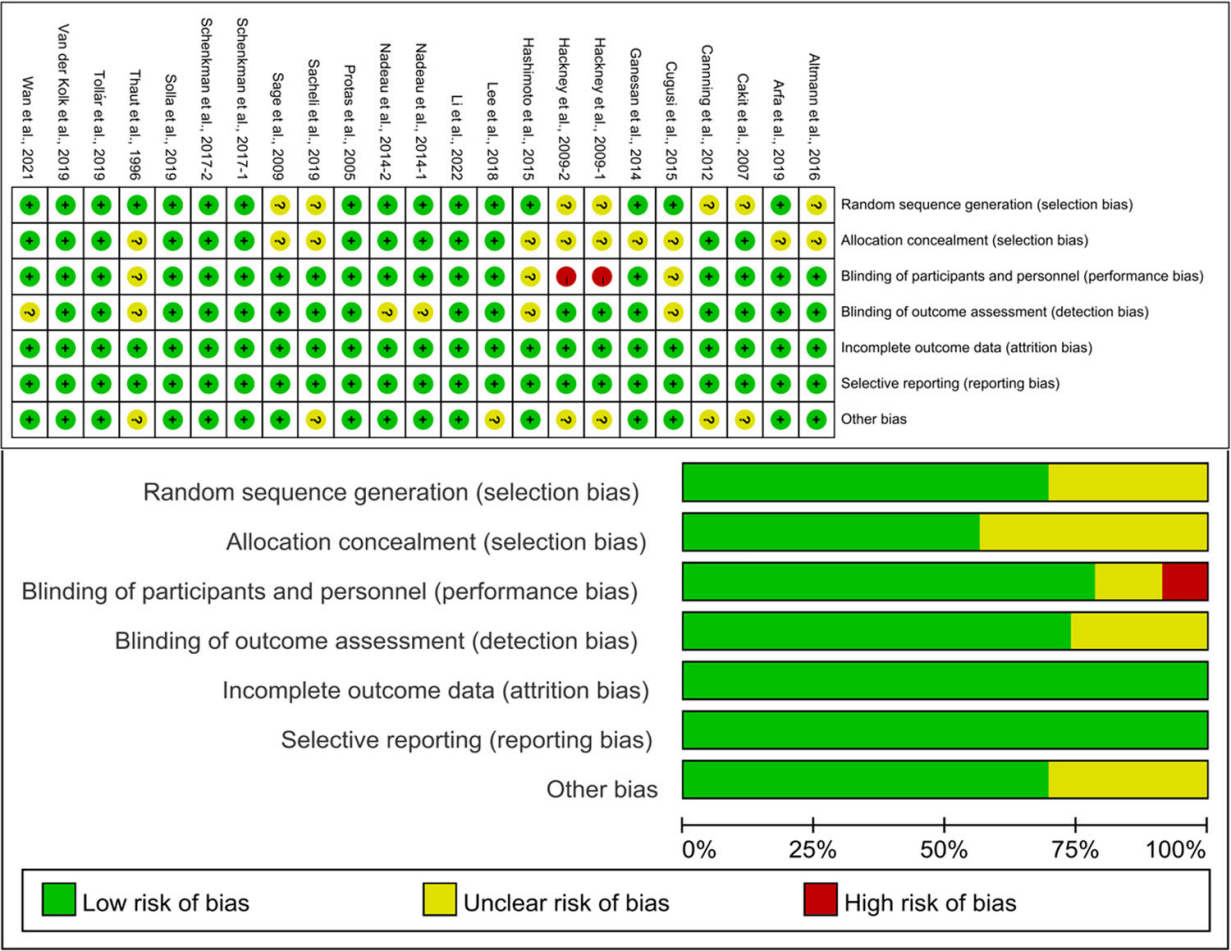
Results of Cochrane risk of bias tool. Above: Risk of bias summary: review authors’ judgments about each risk of bias item for each included study. Below: Risk of bias graph: review authors’ judgments about each risk of bias item presented as percentages across all included studies.

**Fig. 3:**
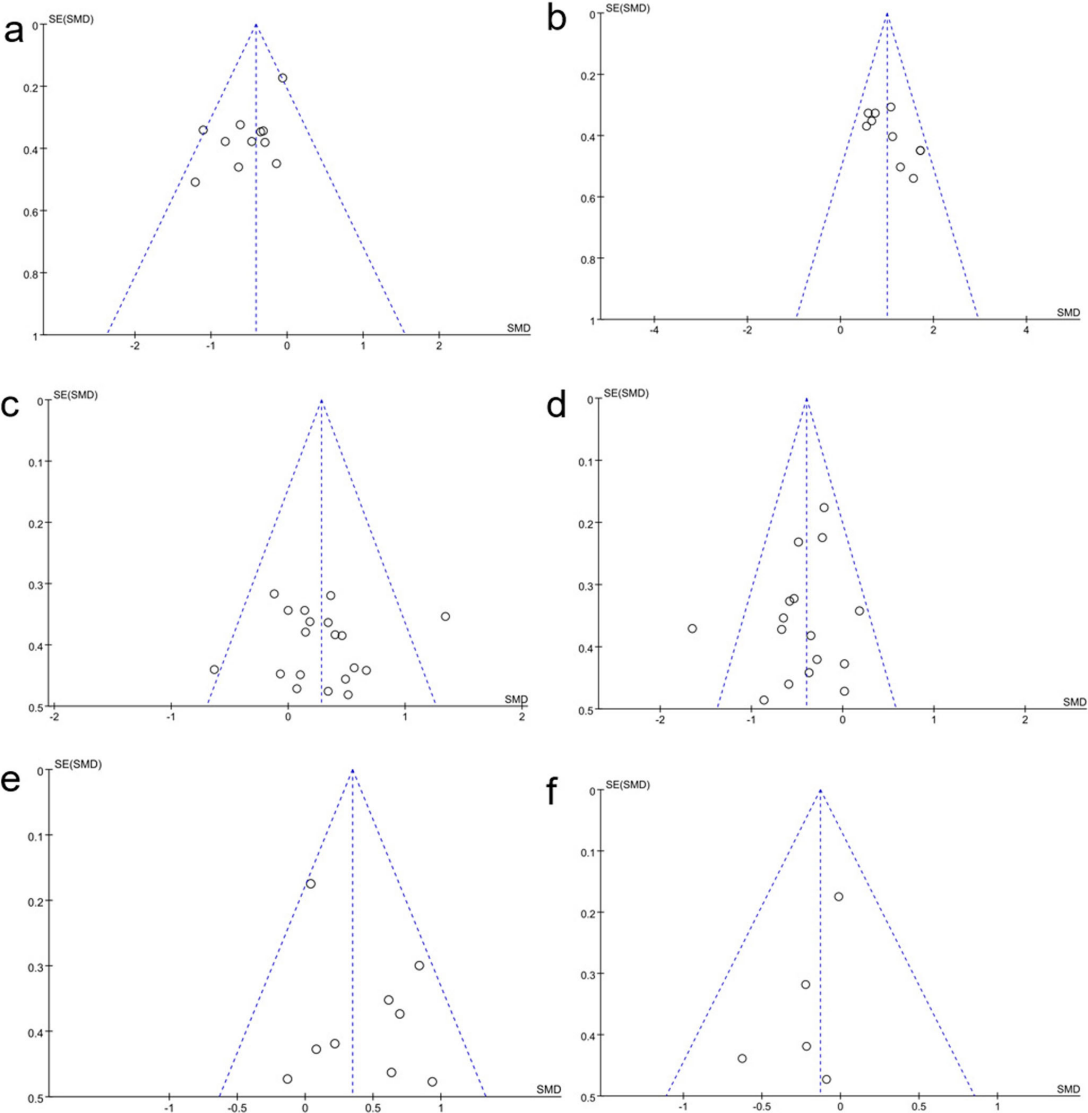
Funnel plot. **a** Funnel plot for TUG. **b** Funnel plot for BBS. **c** Funnel plot for gait. **d** Funnel plot for UPDRS-III. **e** Funnel plot for 6MWT. **f** Funnel plot for PDQ-39.

Cochrane risk assessment tool was used to evaluate the methodological quality of the included literature, mainly from six aspects: selection bias, performance bias, detection bias, attrition bias, reporting bias, and other bias. As shown in Fig. 2, the included studies were graded as low quality, moderate quality, or high quality based on the following criteria: (1) trials were considered low quality if either randomization or allocation concealment was assessed as a high risk of bias, regardless of the risk of other items; (2) trials were considered moderate quality if they did not meet criteria for high or low risk; (3) trials were considered high quality when both randomization and allocation concealment was assessed as a low risk of bias, and all other items were assessed as low or unclear risk of bias in a trial^44^. One study provided evidence of low quality, one study provided evidence of moderate quality, and 18 studies provided evidence of high quality. Publication bias was assessed visually by inspecting the funnel plot (Fig. 3).

### Meta-analysis results

#### Effects of aerobic exercise on TUG in people with PD

**Fig. 4:**
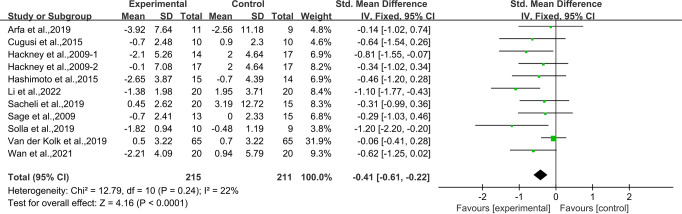
Meta-analysis results of the effect of aerobic exercise on TUG in people with PD. The pooled estimates were obtained from fixed effects analysis. Diamonds indicated the effect size of each study summarized as SMD. The size of the shaded squares was proportional to the percentage weight of each study. Horizontal lines represented the 95% CI and the vertical line represented the overall effect.

Ten studies^24,28,30,31,33–38^ provided data for TUG. It was found that compared with the control group, aerobic exercise had a significant effect on improving TUG in people with PD [SMD, −0.41 (95% CI, −0.61 to −0.22), *p* < 0.00001, *I*^2^ = 22%, Fig. 4].

### Effects of aerobic exercise on BBS in people with PD

**Fig. 5:**
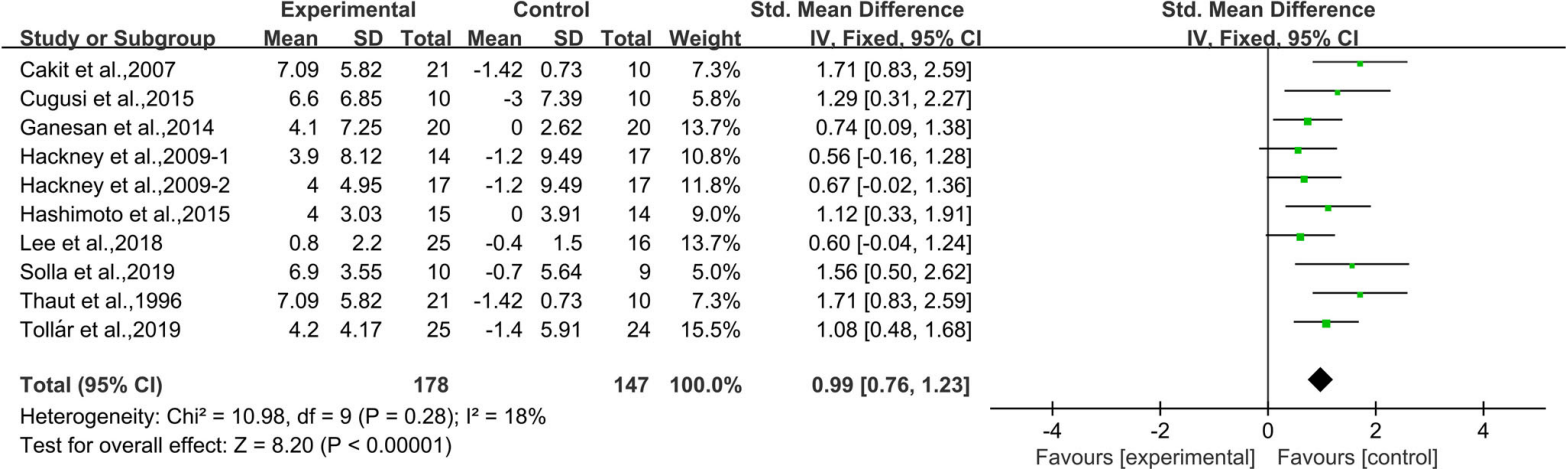
Meta-analysis results of the effect of aerobic exercise on BBS in people with PD. The pooled estimates were obtained from fixed effects analysis. Diamonds indicated the size of the effect of each study summarized as SMD. The size of the shaded square was proportional to the percentage weight of each study. Horizontal lines represented the 95% CI and the vertical dashed line represented the overall effect.

Nine studies^24–32^ provided data for BBS. It was found that compared with the control group, aerobic exercise had a significant effect on improving BBS in people with PD [SMD, 0.99 (95% CI, 0.76 to 1.23), *p* < 0.00001, *I*^2^ = 18%, Fig. 5].

### Effects of aerobic exercise on gait in people with PD

**Fig. 6:**
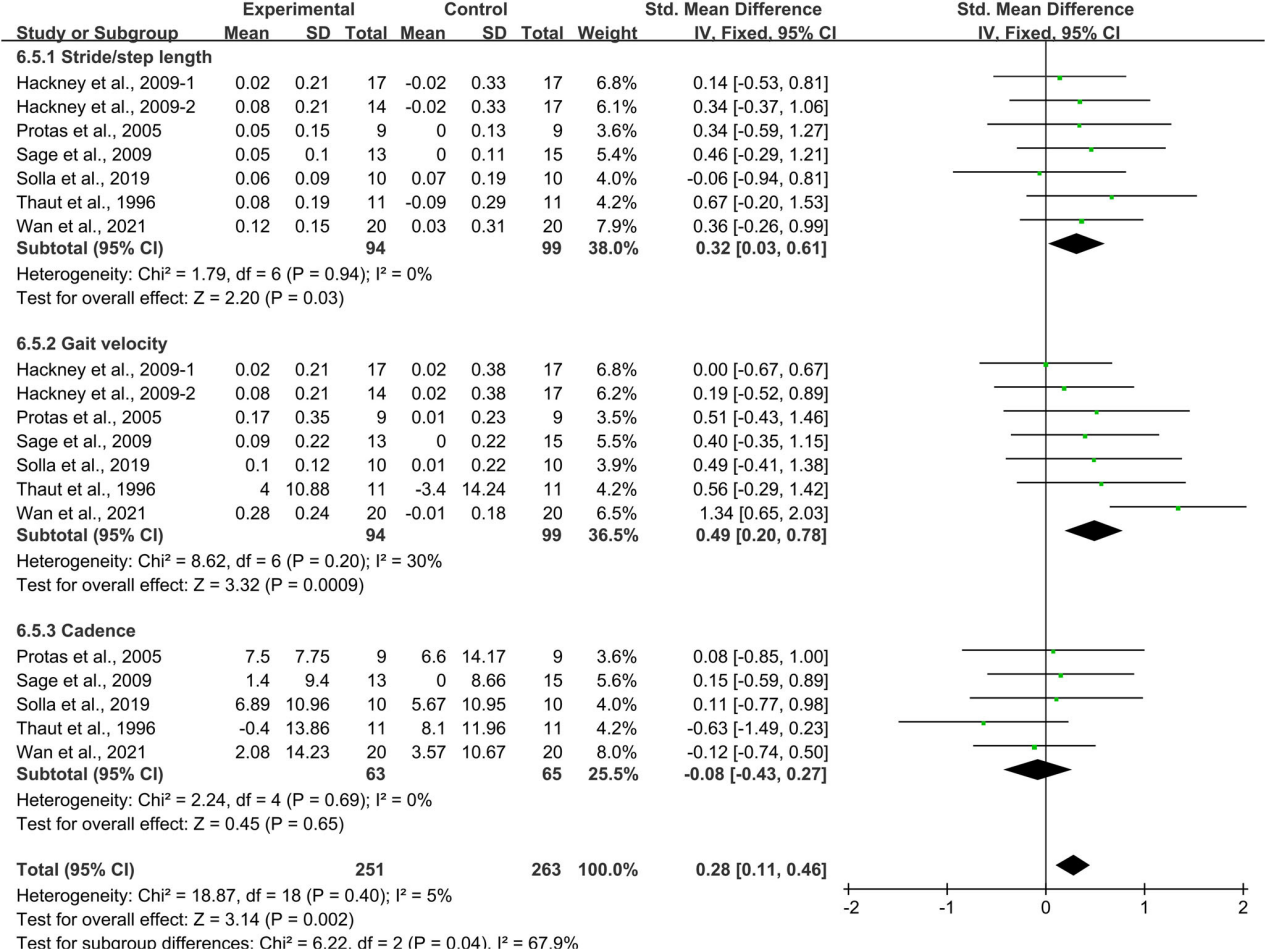
Meta-analysis results of the effect of aerobic exercise on gait in people with PD. The pooled estimates were obtained from fixed effects analysis. Diamonds indicated the size of the effect of each study summarized as SMD. The size of the shaded square was proportional to the percentage weight of each study. Horizontal line represented the 95% CI and the vertical dashed line represented the overall effect.

Six studies^24,25,31,34,36,43^ provided data for stride/step length and gait velocity, and five studies^25,31,34,36,43^ provided data for step cadence. It was found that compared with the control group, aerobic exercise had a significant effect on improving gait in people with PD. Specifically, aerobic exercise significantly increased the stride/step length [SMD, 0.32 (95% CI, 0.03 to 0.61), *p* = 0.03, *I*^2^ = 0%, Fig. 6] and the gait velocity [SMD, 0.49 (95% CI, 0.20 to 0.78), *p* = 0.0009, *I*^2^ = 30%, Fig. 6] in people with PD. However, aerobic exercise had no significant associations with the step cadence in people with PD [SMD, −0.08 (95% CI, −0.43 to 0.27), *p* = 0.65, *I*^2^ = 0%, Fig. 6].

### Effects of aerobic exercise on UPDRS-III in people with PD

**Fig. 7:**
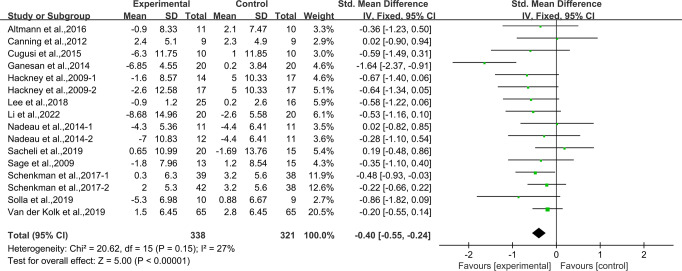
Meta-analysis results of the effect of aerobic exercise on UPDRS-III in people with PD. The pooled estimates were obtained from fixed effects analysis. Diamonds indicated the size of the effect of each study summarized as SMD. The size of the shaded square was proportional to the percentage weight of each study. Horizontal line represented the 95% CI and the vertical dashed line represented the overall effect.

Thirteen studies^24,27–29,31,33,36–42^ provided data for UPDRS-III. It was found that compared with the control group, aerobic exercise had a significant effect on reducing UPDRS-III in people with PD [SMD, −0.40 (95% CI, −0.55 to −0.24), *p* < 0.00001, *I*^2^ = 27%, Fig. 7].

### Effects of aerobic exercise on 6MWT in people with PD

**Fig. 8:**
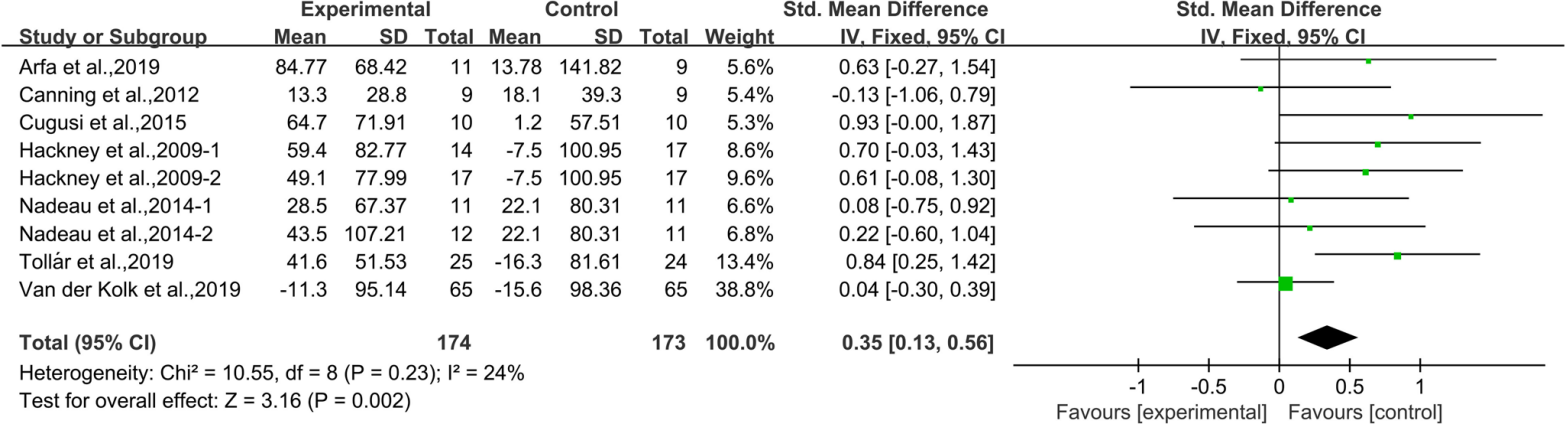
Meta-analysis results of the effect of aerobic exercise on 6MWT in people with PD. The pooled estimates were obtained from fixed effects analysis. Diamonds indicated the size of the effect of each study summarized as SMD. The size of the shaded square was proportional to the percentage weight of each study. Horizontal line represented the 95% CI and the vertical dashed line represented the overall effect.

Eight studies^24,28,31–33,35,39,42^ provided data for 6MWT. It was found that compared with the control group, aerobic exercise had a significant effect on improving 6MWT in people with PD [SMD, 0.35 (95% CI, 0.13 to 0.56), *p* = 0.002, *I*^2^ = 24%, Fig. 8].

### Effects of aerobic exercise on PDQ-39 in people with PD

**Fig. 9:**
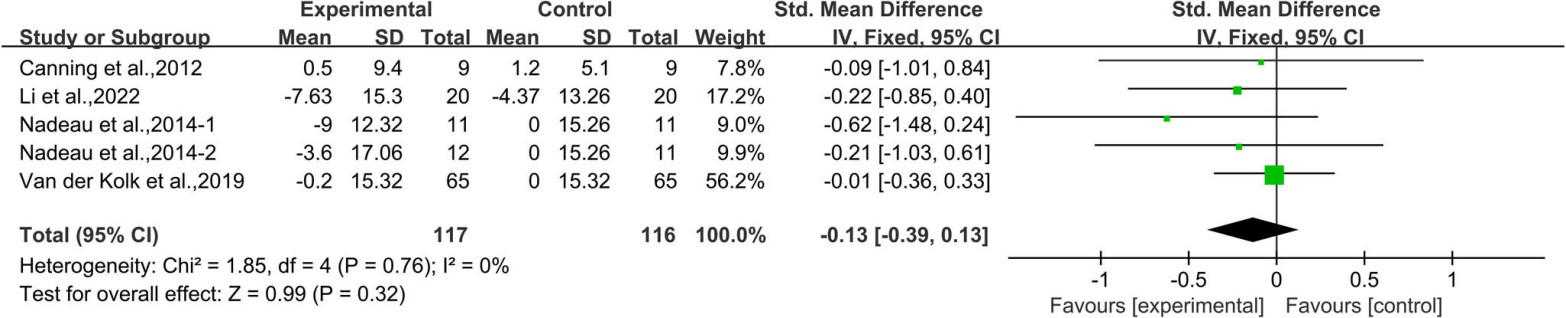
Meta-analysis results of the effect of aerobic exercise on PDQ-39 in people with PD. The pooled estimates were obtained from fixed effects analysis. Diamonds indicated the size of the effect of each study summarized as SMD. The size of the shaded square was proportional to the percentage weight of each study. Horizontal line represented the 95% CI and the vertical dashed line represented the overall effect.

Four studies^33,37,39,42^ provided data for PDQ-39. It was found that aerobic exercise had no significant associations with PDQ-39 in people with PD [SMD, −0.13 (95% CI, −0.39 to 0.13), *p* = 0.32, *I*^2^ = 0%, Fig. 9].

### Adverse events

Adverse events related to exercise intervention were reported for 1 of 20 studies (5.0%). Eleven adverse events were reported. Specifically, seven adverse events occurred in the intervention group and four adverse events occurred in the control group. Further, the majority of adverse events were not associated with the exercise intervention, meaning that aerobic exercise was safe for PD patients.

### Follow-up effect

Three studies reported on follow-up periods of 6 weeks to 6 months. Two studies showed that the effect of aerobic exercise on PDQ-39 was only significantly different at follow-up (6 weeks and 2 months, respectively) compared to post-exercise testing^43,45^. One study reported significant differences in UPDRS-III after 6 months of follow-up, suggesting a persistent effect of aerobic exercise on motor symptoms in PD patients^43^.

## Discussion

Twenty studies examining the effect of aerobic exercise on balance, gait, motor function, and quality of life in PD patients were considered eligible for systematic review and meta-analysis. However, the included studies were all RCTs of aerobic exercise intervention, which could not be completely blinded. Previous study has shown that results of a trial using the best possible methods may still be at risk of bias. For example, blinding may not be feasible in many non-drug trials, and it would not be reasonable to consider the trial as low quality because of the absence of blinding^44^. Therefore, in the quality evaluation process, 18 studies were considered to be of high quality, which contributed to strengthen our results and conclusions.

This systematic review and meta-analysis indicated that aerobic exercise had the potential to improve balance, gait (velocity and stride/step length), and motor function in people with PD compared to placebo or no intervention. In addition, our results showed no significant heterogeneity in TUG (*I*^2^ = 22%), BBS (*I*^2^ = 18%), stride/step length (*I*^2^ = 0%), step cadence (*I*^2^ = 0%), 6MWT (*I*^2^ = 24%), and PDQ-39 (*I*^2^ = 0%), and low heterogeneity in UPDRS-III (*I*^2^ = 27%) and gait velocity (*I*^2^ = 30%), indicating consistent results from the included studies. Therefore, we did not perform subgroup analysis or sensitivity analysis. However, there was insufficient data to support the effectiveness of aerobic exercise in improving quality of life after post-exercise testing, but two studies^40,43^ showed improved quality of life in PD patients at 2-week and 2-month follow-up, respectively, which suggested that the effect of aerobic exercise on the quality of life of PD patients may take a long time to be observed.

Most people with PD suffer from impairments in maintaining a balanced and stable posture. Sometimes, they fall, which can be dangerous in normal life. Statistics showed that falls affect more than 50% of PD patients^46,47^. BBS and TUG are commonly used to assess posture balance and control^39,48^. Our study provided evidence that aerobic exercise improved BBS and TUG in PD patients, as well as enhanced physical control and falls prevention in daily life, which was consistent with a previous study, showing that the benefits of Nordic walking lie in improved coordination between the upper and lower limbs, resulting in an improved balance in daily living tasks with greater complexity^49^.

Creaby et al.^46^ emphasized that PD patients have a slower gait velocity and are at greater risk of falls and complications. Therefore, improving gait is important for preventing falls in PD patients. Our meta-analysis showed that aerobic exercise had a positive effect on gait velocity and stride/step length, while aerobic exercise had no significant associations with step cadence in people with PD, which was consistent with previous studies, showing that 4 to 24 weeks of aerobic exercise significantly improved both gait velocity and stride/step length^39,47–49^. Aerobic exercise can promote postural responses and decoupling of pelvis and shoulder girdle movements, thereby reducing axial stiffness. Improvements in trunk control may contribute to improvements in balance, walking, and posture^50,51^. However, there is not much evidence to support the effect of aerobic exercise on step cadence. Nadeau et al.^39^ reported that a longer aerobic exercise on a treadmill (24 months) was able to promote improvements in step cadence, while a shorter protocol (4 months) did not achieve this benefit. Therefore, the effect of aerobic exercise intensity and duration on gait are unclear and more research is needed.

Motor symptoms of PD include tremor, bradykinesia, hypokinesia, bradykinesia, rigidity, gait disturbances, and postural disturbances. The severity of the motor signs is usually assessed by UPDRS-III^52^. Dyskinesia progresses rapidly, with UPDRS-III scores ranging from 1.5 to 8.9 per year^53,54^. In our study, aerobic exercise had a significant effect on reducing UPDRS-III in people with PD, which was consistent with a previous meta-analysis, showing that aerobic exercise reduced UPDRS-III significantly^55,56^. Previous studies revealed that aerobic walking was safe, well-tolerated, and improved aerobic fitness, motor function, and quality of life in people with mild to moderate PD^57,58^. Changes in UPDRS-III demonstrate the positive effects of aerobic intervention, as UPDRS is considered the gold standard for such analyses^59^. Carvalho et al.^60^ showed that both aerobic exercise and strength training improved motor symptoms in PD patients (UPDRS-III was 35 and 27.5%, respectively), while aerobic exercise was better.

The 6MWT provides a measure of walking endurance^61^. The test was conducted in a 100-m long corridor where participants were asked to walk as fast as possible for 6 min, and the total walking distance (m) was measured by the evaluator. Our meta-analysis showed that aerobic exercise had a positive effect on improving 6MWT in people with PD, which was consistent with a previous study, showing that walking improved regardless of the duration of aerobic exercise^58^. In addition, studies have shown that aerobic exercise improved the ability of PD patients to deal with behaviorally complex motor tasks, while aerobic exercise also had a positive effect on enhancing balance function and walking ability^58,61,62^.

The PDQ-39 has 39 items, designed by Peto et al.^63^. The higher the score, the lower the quality of life. The PDQ-39 has eight subscales, namely mobility (ten items), activities of daily living (six items), emotional well-being (six items), stigma (four items), social support (three items), cognition (four items), communication (four items), and bodily discomfort (three items)^64–67^. PDQ-39 is currently recognized as the most comprehensive and widely used specific scale for evaluating the quality of life of PD patients^65^. However, most studies have shown no significant improvement in the quality of life of PD patients after aerobic exercise intervention, which was consistent with our study. de Oliveir et al.^68^ conducted a meta-analysis including ten studies with 411 PD patients and concluded that there was no significant improvement in PDQ-39 after aerobic exercise compared to the usual care or placebo group. However, Chen et al.^69^ reported that aerobic exercise could improve the quality of life in PD patients, but different scales such as the PDQ-39 scale, Parkinson’s disease quality of life questionnaire (PDQL), EuroQol (EQ-5D) and other types of scales were used to assess the quality of life. Therefore, since different scales have different criteria, there may be evaluation bias. It has been reported that PD patients are better able to concentrate, remember, and recall information after the aerobic intervention, which may have a positive impact on quality of life^70^. In this meta-analysis, studies that met the inclusion criteria reported limited data on quality of life. Therefore, more research on the quality of life of PD patients using the same scale should be conducted, and data collection and research analysis should be emphasized in the future.

PD is a progressive neurodegenerative disease caused by the progressive loss of neurons and protein deposition in the brain that exhibits altered physicochemical properties^71–73^. Aerobic exercise has been associated with neuroprotective effects in the nigrostriatal dopaminergic system, so as to improve many motor and non-motor Parkinson’s symptoms, and the proposed mechanisms that link physical activity to neuroprotective effect are an increase in serum urate, an increased release of neurotrophic factors [e.g., brain-derived neurotrophic factor (BDNF) and glial cell-derived neurotrophic factor (GDNF)], upregulation of the transcriptional regulator peroxisome proliferator-activated receptor gamma (PPARγ) coactivator 1α (PGC1α), and regulation of dopamine turnover^74^. A peripheral indicator of neurogenesis is BDNF, which helps to support neuronal growth and survival^71–73^. Decreased levels of neurotrophic factors, mainly BDNF and its receptors, are among the most common physiological changes in neurodegenerative diseases such as PD^75–78^. A previous study has shown that aerobic exercise induced an increase in BDNF levels, protected dopaminergic neurons, and was involved in the activation and recovery of motor function^79^. In addition, Zoladz et al.^80^ also reported that aerobic exercise can be therapeutically effective by increasing BDNF levels. Previously, it was suggested that aerobic intervention lasting at least 12 weeks for a duration of 40 min is the most effective exercise strategy for increasing BDNF levels^81^. A meta-analysis also showed that aerobic exercise increased BDNF levels in people with neurological disorders compared to the usual care or placebo group. Upregulation of BDNF is considered desirable because it is associated with enhanced plasticity-related processes such as dendritic growth, neurogenesis, and long-term potentiation of neurons^82^.

Furthermore, aerobic exercise, as an effective non-drug therapy, not only promotes physical fitness in PD patients, but also improves motor learning in daily activities by enhancing the plasticity of exercise-related structures^83^. Rosenfeldt et al.^84^ reported that 8 weeks of aerobic exercise altered various signaling pathways in the central nervous system, modulated cognitive or physiological processes, and controlled the sense of smell in PD patients. Retrospective studies have also found that moderate-intensity to vigorous-intensity aerobic exercise can prevent PD^85,86^. Aerobic exercise is a very popular functional recovery treatment that has positive effects on motor function, quality of life, cognition, and mood in PD patients. Therefore, functional improvements, motor symptoms, balance, and gait improvements can be explained by the cellular and biochemical responses induced by aerobic exercise, which acts as a regulator of neurogenesis and neuronal plasticity and is neuroprotective in various cortical regions^87–90^.

This systematic review and meta-analysis also had several limitations that should be noted. Firstly, the included studies were all RCTs of aerobic exercise intervention, which could not be completely blinded. Therefore, in the quality evaluation process, subjective factors will cause a certain degree of deviation. Secondly, the scale for assessing the quality of life in patients with PD is not unique, we chose PDQ-39 as a data source, resulting in fewer data included. There may be some bias in assessing the impact of aerobic exercise on quality of life. Finally, we did not analyze the longer-term effect of aerobic exercise on PD as only a few included studies reported follow-up data in the meta-analysis.

Our analysis indicated that aerobic exercise had beneficial effects in improving balance, gait (velocity and stride/step length), and motor function in PD patients. However, aerobic exercise had no significant associations with the step cadence and quality of life in PD patients. Therefore, in future studies, these findings should shed new light on the very important issue that aerobic exercise may improve balance, gait, and motor function in patients with PD.

## Method

This systematic review and meta-analysis were conducted following the guidelines of the Cochrane Selection Manual^91^ and the Preferred Reporting Items for Systematic Reviews and Meta-Analysis (PRISMA)^92^. The protocol for this systematic review has been registered on PROSPERO (CRD42022340730).

### Search strategy

For this systematic review and meta-analysis, we searched through PubMed, Web of Science, and EBSCO electronic databases from the inception of indexing until September 5, 2022. All studies on aerobic exercise and PD were searched using the following MESH terms and keywords: aerobic exercise and Parkinson’s disease. We also hand-searched reference lists of all identified studies. We excluded studies based on the review of the title, abstract, and full text. Two authors (KZ and SZ) conducted the process independently using a standardized form. In case of any discrepancies between the two authors, a third author (LY) was involved in the discussion until a consensus was made.

### Eligibility criteria

We included studies that satisfied the following criteria: eligible studies (1) should be RCTs; (2) should include both an intervention and control group with the only difference between them being the addition of aerobic exercise in the intervention group; (3) should use PD patients as subjects; and (4) should use balance, gait, motor function, or quality of life as the outcome measure. Non-English language publications, animal model publications, reviews, and conference articles were excluded from the analysis.

### Data extraction

Two authors of the review performed the data extraction independently using the same standardized form created in Microsoft Excel. If there were any discrepancies between the authors in the extracted data, the accuracy of the information was re-checked in the studies. The extracted variables mainly included: (a) characteristics of included studies (first author’s last name, year of study publication); (b) characteristics of aerobic exercise (intensity, session duration, frequency); (c) participant’s characteristics (*n*, stage of disease, disease duration); (d) treatment effects [mean and standard deviation (SD) values reflecting the change in timed up and go test (TUG), Berg balance scale (BBS), stride/step length, step cadence, unified Parkinson’s disease rating scale part-III (UPDRS-III), 6 min walking test (6MWT), and Parkinson’s disease questionnaire-39 (PDQ-39) from baseline and to post-intervention in the aerobic exercise and control groups.

### Methodological quality assessment

We assessed the methodological quality of the included studies using the Cochrane risk of bias criteria, which included seven items: randomization sequence generation (selection bias), allocation concealment (selection bias), blinding of participants and personnel (performance bias), blinding of outcome assessment (detection bias), incomplete outcome data (attrition bias), selective reporting (reporting bias), and other bias. Each item was judged as either “low risk”, “unclear risk”, or “high risk” based on responses to the signaling questions, to make an overall bias judgment for the specific study outcome being assessed^91,93^. Two reviewers performed the methodological quality assessment independently. Disagreements in the assessments between the reviewers were resolved through discussion and consensus with a third author.

### Statistical analysis

The mean and SD values reflecting the change in TUG, BBS, balance, UPDRS-III, 6MWT, and PDQ-39 from baseline and to post-intervention were extracted from each study for pooling effects. SD was calculated using a previously described formula for studies reporting standard error (SE) or 95% confidence intervals (CIs)^94^. When the data could not be extracted or there was a dispute, two authors negotiated or contacted the author of the article to resolve it. Data were pooled using fixed effects models to obtain the standardized mean difference (SMD) and 95% CIs. Heterogeneity was assessed by Cochrane’s Q and *I*^2^ static. *I*^2^ < 25% indicates no significant heterogeneity; 25% < *I*^2^ < 50%, low heterogeneity; 50% < *I*^2^ < 75%, medium heterogeneity; *I*^2^ > 75%, high heterogeneity. If there was a high level of heterogeneity in the test, we used subgroup analysis or sensitivity analysis to explain the results. The analysis result, funnel plot, and forest chart were generated using the software RevMan.5. In terms of overall impact, *p* < 0.05 were considered statistically significant.

### Reporting summary

Further information on research design is available in the Nature Research Reporting Summary linked to this article.

## Supplementary information

Reporting Summary

## Data Availability

All data generated or analysed during this study are included in this published article.
